# Exploring the relevance of *NUP93* variants in steroid-resistant nephrotic syndrome using next generation sequencing and a fly kidney model

**DOI:** 10.1007/s00467-022-05440-5

**Published:** 2022-02-24

**Authors:** Agnieszka Bierzynska, Katherine Bull, Sara Miellet, Philip Dean, Chris Neal, Elizabeth Colby, Hugh J. McCarthy, Shivaram Hegde, Manish D. Sinha, Carmen Bugarin Diz, Kathleen Stirrups, Karyn Megy, Rutendo Mapeta, Chris Penkett, Sarah Marsh, Natalie Forrester, Maryam Afzal, Hannah Stark, NIHR BioResource, Maggie Williams, Gavin I. Welsh, Ania B. Koziell, Paul S. Hartley, Moin A. Saleem

**Affiliations:** 1 Bristol Renal and Children’s Renal Unit, Bristol Medical School, University of Bristol, Whitson Street, Bristol, BS1 3NY UK; 2 Nuffield Department of Medicine, University of Oxford, Oxford, UK; 3 Department of Life and Environmental Science, Bournemouth University, Talbot Campus, Fern Barrow, Poole, Dorset BH12 5BB England, UK; 4 Illawarra Health and Medical Research Institute, Molecular Horizons and School of Medicine, University of Wollongong, Wollongong, Australia; 5 Bristol Genetics Laboratory, North Bristol National Health Service Trust, Bristol, UK; 6 School of Medicine, University of Sydney and Children’s Hospital at Westmead, Westmead, Australia; 7 Children’s Kidney Centre, University Hospital of Wales, Cardiff, UK; 8 Department of Paediatric Nephrology, Evelina London Children’s Hospital, Guy’s and St, Thomas’ Hospital, London, UK; 9 School of Immunology and Microbial Sciences, Faculty of Life Sciences and Medicine, King’s College London, London, SE1 7EH UK; 10 NIHR BioResource, Cambridge University Hospitals, Cambridge Biomedical Campus, Cambridge, CB2 0QQ UK; 11 Centre for Genomics Research, Discovery Sciences, BioPharmaceuticals R&D, AstraZeneca, Cambridge, UK; 12 Wellcome Sanger Institute, Wellcome Genome Campus, Hinxton, Cambridge, CB10 1SA UK

**Keywords:** *NUP93*, SRNS, FSGS, Podocyte, Nephrocyte

## Abstract

**Background:**

Variants in genes encoding nuclear pore complex (NPC) proteins are a newly identified cause of paediatric steroid-resistant nephrotic syndrome (SRNS). Recent reports describing *NUP93* variants suggest these could be a significant cause of paediatric onset SRNS. We report *NUP93* cases in the UK and demonstrate in vivo functional effects of *Nup93* depletion in a fly (*Drosophila melanogaster)* nephrocyte model.

**Methods:**

Three hundred thirty-seven paediatric SRNS patients from the National cohort of patients with Nephrotic Syndrome (NephroS) were whole exome and/or whole genome sequenced. Patients were screened for over 70 genes known to be associated with Nephrotic Syndrome (NS). *D. melanogaster Nup93* knockdown was achieved by RNA interference using nephrocyte-restricted drivers.

**Results:**

Six novel homozygous and compound heterozygous *NUP93* variants were detected in 3 sporadic and 2 familial paediatric onset SRNS characterised histologically by focal segmental glomerulosclerosis (FSGS) and progressing to kidney failure by 12 months from clinical diagnosis.

Silencing of the two orthologs of human *NUP93* expressed in *D. melanogaster*, *Nup93-1*, and *Nup93-2* resulted in significant signal reduction of up to 82% in adult pericardial nephrocytes with concomitant disruption of NPC protein expression. Additionally, nephrocyte morphology was highly abnormal in *Nup93-1* and *Nup93-2* silenced flies surviving to adulthood.

**Conclusion:**

We expand the spectrum of *NUP93* variants detected in paediatric onset SRNS and demonstrate its incidence within a national cohort. Silencing of either *D. melanogaster Nup93* ortholog caused a severe nephrocyte phenotype, signaling an important role for the nucleoporin complex in podocyte biology.

**Graphical Abstract:**

A higher resolution version of the Graphical abstract is available as Supplementary information 
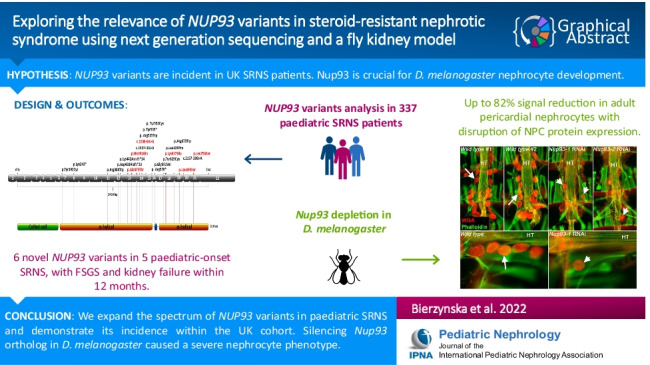

**Supplementary Information:**

The online version contains supplementary material available at 10.1007/s00467-022-05440-5.

## Introduction

Nuclear pore complexes (NPCs) are channels composed of evolutionarily conserved proteins called nucleoporins (NUPs), penetrating the nuclear envelope to tightly regulate transport of proteins and RNA between the nucleoplasm and cytoplasm [1]. Multiple copies of about 30 NUPs arranged around the central transport channel form each NPC. In vertebrates this structure is pre-assembled into a core scaffold made of the NUP93/NUP205 complex and two rings formed by the NUP107/NUP160 complex [2]. Additional roles for NUP proteins are emerging, beyond nucleocytoplasmic transport, including transcriptional regulation, DNA damage response and chromosome segregation in mitosis [2]. NUP93 in particular has been linked to ciliary function and cell type specific regulation of gene expression [3, 4]. Depletion of NUP93 impairs correct assembly of the nuclear pore [5, 6].

NPC composition varies among cell types, with tissue-specific transport functions and regulatory roles [7] and mutations previously linked to phenotypes such as atrial fibrillation [8] and leukaemia [9]. In the kidney, NUP93 is expressed in all kidney cell types. As such, *NUP93* mutations reduce signal intensity in both glomerular and extra-glomerular cells of kidney cortex [10]. However, in vitro knockdown of *NUP93* in human podocytes reduces cell migration, proliferation rate and expression of NUP205, whereas overexpression of SRNS-associated *NUP93* variants in *NUP93*-deleted *X.laevis* egg extracts lead to disrupted assembly of the NPC [11]. *NUP93* mutations also abolish protein–protein interaction with SMAD4 and importin 7 potentially disrupting nucleocytoplasmic trafficking [11]. Equally, RNAi depletion of *NUP93* in *C.elegans* in vivo results in atypical NPC distribution within the nuclear envelope and failure in nuclear exclusion of non-nuclear macromolecules [12]. No in vivo model of *NUP93* knockdown has yet been described that disturbs podocyte or glomerular function.

*D. melanogaster* nephrocytes are podocyte-like and situated pericardially, adjacent to the heart tube. Analogous to mammalian podocytes, insect nephrocytes also form slit diaphragms [13, 14] with evolutionary conservation of many of the slit diaphragm proteins making *D.melanogaster* an important animal model of human podocyte biology [15–17]. Two orthologues of human NUP93, Nup93-1 and Nup93-2, are expressed; while NUP93-2 has widespread tissue expression, NUP93-1 is virtually specific to pericardial nephrocytes [18].

Mutations in 7 nuclear pore associated genes, *NUP85, NUP107, NUP133, NUP160, NUP205, XP05* and *NUP93* have recently been implicated in paediatric SRNS forming the first kidney-specific example of NPC dysfunction [11, 19, 20]. To date, 15 different *NUP93* mutations are reported in 16 SRNS patients with the most common variant, p.(Gly591Val), found in 9/16 cases [11, 21–24]. We recently described a UK cohort of paediatric SRNS patients, collected via a national UK Renal Registry (RaDaR) [25]. Exome sequencing was performed on the first 187 patients, with an additional 150 patients also whole genome sequenced. All patients were initially screened for mutations in the 76 genes currently known to be associated with NS (Supplementary Table 1).

We were surprised to find a higher-than-expected incidence of novel or very rare variants in *NUP93* in 4 unrelated pedigrees within this relatively small paediatric cohort compared with that of other previously-described podocyte gene mutations*.* As such, we expand the description of *NUP93* variants in SRNS, and our experimental results provide the first in vivo evidence that NPC disruption, resulting from *NUP93-1* and/or *NUP93-2* knockdown in the *D. melanogaster* nephrocyte, significantly affects glomerular filtration barrier function.

We propose that this in vivo model has the potential to serve as a gold standard in the future for functional assay of model single missense variants in *NUP93* to establish likely pathogenicity.

## Material and methods

### Sequencing

Patient 85 was whole exome sequenced as previously described [25]. Patient 7 tested negative for 24 genes associated with SRNS [26]*,* so was subsequently whole genome sequenced within the WGS500 project (Oxford Genomics Centre at the Wellcome Centre for Human Genetics) [27]. His sister, 7S, was screened at the Bristol Genetics Laboratory to confirm the gene variant (SRNS gene panel, www.nbt.nhs.uk/severn-pathology/pathology-services/bristol-genetics-laboratory-bgl). Patients S013282 and S013682 were whole genome sequenced at the NIHR BioResource (Cambridge, UK) [28]. Sequencing was performed on an Illumina platform with mean coverage of 35X. Variant calling and annotation were performed using the King’s College London BRC Genomics [25] and NIHR BioResource [28] pipelines. All detected variants were filtered and classified as described previously [25, 29] and the description of each step is described in the Supplemental Data.

Homozygosity mapping [30] was performed for patient S013682. All homozygous variants found within the coding sequence and the detected stretches of homozygosity were analysed. Variants with MAF > 0.01 or seen as homozygotes in gnomAD database or an in-house control database or affecting a non-conserved amino acid were filtered out. Synonymous variants were ignored since they are less likely to affect the protein.

### Animal model—NUP93 knock-down in Drosophila

Flies were raised on a standard cornmeal-yeast diet at 25 °C under a 12:12 h light:dark cycle. Knockdown was achieved by RNA interference using the following fly strains: *UAS-NUP 93–2* RNAi (Bloomington stock centre, number 51758,); *UAS-NUP93-1* (Bloomington stock centre, number 34090, both Harvard TRiP lines containing a targeting hairpin insertion in the third chromosome [31], *DotGal4*[32] and *dKlf15Gal4* [33].

### Staining of adult Drosophila nephrocytes

For staining, 4–9 day old flies were dissected as previously described [34]. In brief, dissected hearts and nephrocytes were fixed in 3.8% formaldehyde in PBS for 20 min, washed 3 × with PBS and then incubated in wheat germ agglutinin (conjugated to Alexa 594 fluorochrome at 1 ug/ml) and Alexa-Fluor-488 conjugated Phalloidin (1/250) to stain F-actin. To stain the NPC, samples were incubated in primary antibody (Mab414-Abcam) and Hoechst to visualise DNA followed by incubation with relevant secondary antibody (Invitrogen, UK). Images were taken using a Zeiss Axiolab widefield LED fluorescent microscope with water dipping × 10 or × 40 objectives. Micrographs were collected using Micromanager and contrast-enhanced using Photoshop, all images being treated equally.

### Statistics

One-way ANOVA followed by Tukey’s post hoc HSD test.

## Results

Four patients from the UK paediatric cohort of NS were found to have rare, highly conserved and predicted novel possibly pathogenic variants in the *NUP93* gene. One patient was found to have a novel variant but of unknown significance (VUS) (Table 1).

**Table 1:** *NUP93* mutations

Patient #	Nucleotide change	Amino acid change	Amino acid conservation to species	SIFT	PolyPhen	MutationTaster	REVEL	CADD PHRED	EVE Score	EVE Class (75% Set)	gnomAD AF (AC/NH/AN)	TWINSUK AF (AC)	Variant Significance
388	c.2084 T > C	p.Leu695Ser	*C. elegans*	D	PosD	DC	0.649	29.2	0.951	P	0.00003536 (10/0/282796)	0.000269687 (1)	Pos. p
	c.2267 T > C	p.Leu756Ser	*C. elegans*	D	PrD	DC	0.662	31	0.824	P		0.000269687 (1)	Pos. p
278, 5000	c.1423G > A (H)	p.Ala475Thr	Baker's yeast	D	PrD	DC	0.741	29.3	0.982	P			Pos. p
S013682	c.1909A > G (H)	p.Lys637Glu	Fruitfly	T	PosD	DC	0.372	24.8	0.332	B			VUS
S013282	c.1473 T > G	p.His491Gln	Baker's yeast	D	PrD	DC	0.731	22.5	0.849	P	0.000004196 (1/0/238330)		Pos. p
	c.1538-6A > G							15.47					Pos. p

*H*, homozygous; gnomAD, v2.1.1 (The Genome Aggregation Database, http://gnomad.broadinstitute.org/ [35]; *AF*, allele frequency; *AC*, allele count; *AH*, number of homozygotes; *AN*, allele number; *D*, deleterious; *LP*, likely pathogenic; *m*, maternal; *T*, tolerated; *PosD*, possibly damaging; *PrD*, probably damaging; *DC*, disease-causing; *VUS*, variant of unknown significance.

*NUP93* transcript used—NM_014669.4, for splice predictions see Figures S1 and S2.

Tools used: Alamut Visual version 2.15 (SOPHiA GENETICS, Lausanne, Switzerland) (including amino acid conservation, SIFT, MutationTaster); Ensembl Variant Effect Predictor (VEP) (PolyPhen, REVEL score, CADD PHRED score, TWINSUK_AF and AC) [52]; EVE score and EVE Class [43]—patient #7 in McCarthy et al. [26]; patient 7 and 7S are siblings; patient 85 included in Bierzynska et al. [25]; patient S013682 included in Turro et al. [28]

Two heterozygous *NUP93* variants were detected in sporadic patient 85. Mother was heterozygous for p.(Leu695Ser); paternal DNA was not available. Both affected amino acids are highly conserved, and in silico tools predicted deleteriousness.

Siblings 7 and 7S shared a novel, homozygous missense p.(Ala475Thr) variant; parents were consanguineous. This variant affects a highly conserved alanine and was predicted to be deleterious.

A homozygous novel p.(Lys637Glu) variant was detected in a second consanguineous case, S013682. Parents were heterozygous for the same variant as was an unaffected maternal uncle. This variant affects a highly conserved amino acid, and the substitution is predicted to be deleterious in silico by 3 out of 5 of the tools used. Homozygosity mapping was performed for additional confirmation. This was able to verify whether the variant falls within a homozygosity run and to test whether there are any alternative variants of interest in this patient. Only 4 homozygous variants were left after the filtering, with p.(Lys637Glu) being the strongest candidate to be associated with the phenotype in this patient (Supplemental Fig. 3 and Table 2).

**Table 2:** Clinical details per patient

Patient #	Sex	Eth	Age of onset (yr)	Parental consang.	Sporadic/ Familial	Steroid resistance	Histology	Length of follow up	Time to kidney failure (mo)	Post Tx recurrence?	Extra Phenotype
7	M	SA	1	Yes	F	Primary	c. FSGS	7 yr (Died at 8yr1mo-Infection, probably CMV pneumonia)	12	No	Unknown
7S	F	SA	1	Yes	F	Presumed	FSGS	3.5 yr	6	No	Microscopic haematuria
85	F	C	6	No	S	Primary	FSGS	6 yr	2	No	Microscopic haematuria
S013682	M	SA	2	Yes	S	Primary	FSGS	15.5 yr	7	Yes	Microscopic haematuria
S013282	M	C	8	No	S	Primary	FSGS	5.5 yr	12	No	Autism spectrum disorder, microscopic haematuria

*Eth*, ethnicity; *M*, male; *F*, female; *SA*, South Asian; *C*, Caucasian; *F*, familial; *S*, xporadic; *consang*., consanguinity; *mo*, months; *yr*, years; *Tx*, transplanted; *HD*, Haemodialysis; *FSGS*, focal segmental glomerulosclerosis; c. FSGS, collapsing Focal segmental.

Patient 7 previously described in McCarthy et al. [26]. 7S – Sister of patient 7; patient 85 previously described in Bierzynska et al. [25]; patient S013682 included in Turro et al. [28]

Two heterozygous variants in trans, inherited from each nonconsanguineous parent, were detected in S013282. The first variant, c.1538-6A > G, located within the consensus sequence in intron 13 was predicted to create an alternative/new acceptor site (Supplemental Fig. 1). The second variant, p.(His491Gln) was predicted deleterious, affecting a highly conserved amino acid. Additionally, this variant was predicted to create a new donor and a new acceptor site (Supplemental Fig. 2).

**Fig. 1:**
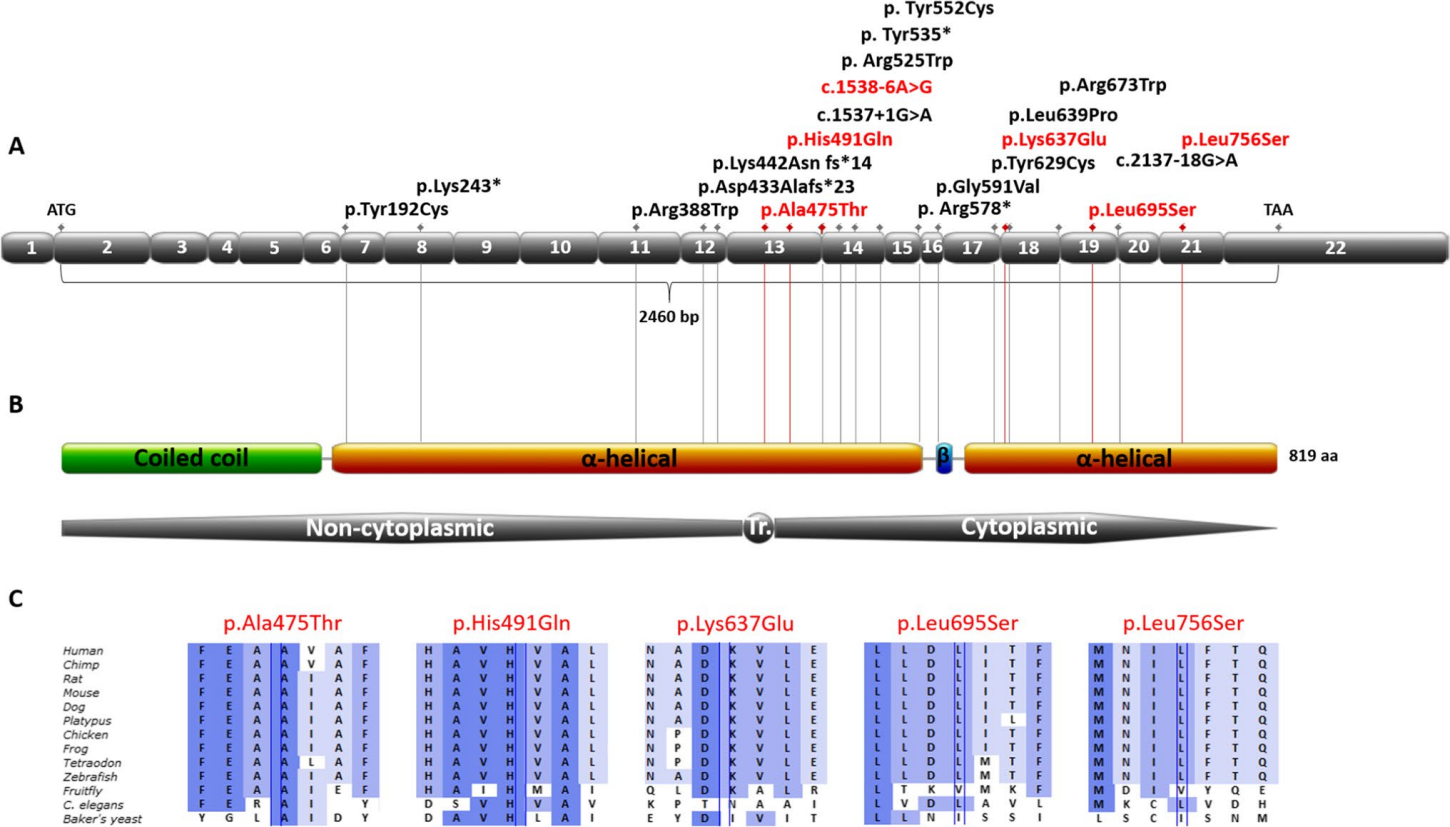
*NUP93* variants A. Exon structure of human *NUP93* cDNA with identified variants. In red: variants found in the UK SRNS cohort, in black: mutations previously described in the literature [10, 11, 21–24, 39, 40]. *NUP93* domain structure created with Prosite MyDomains (https://prosite.expasy.org/cgi-bin/prosite/mydomains) [51]. B. NUP93 protein domain structure with variant positions indicated. Tr, transmembrane; C, conservation across evolution of altered amino acids for the 5 missense variants. Alamut Visual version 2.15 (SOPHiA GENETICS, Lausanne, Switzerland)

Four of the *NUP93* variants detected are not present in gnomAD and the remaining 2 are rare with a very low minor allele frequency (MAF) at 0.00003536 (counts:10/0/282796) and 0.000004196 (counts:1/0/238330) and not reported as homozygotes [35]. All 6 *NUP93* variants are located in the second part of the gene, similar to the previously described mutations [10, 11, 21–23], and are within the 2 α-helical domains of Nup93 (Fig. 1). Location of the variants on the predicted human 3D NUP93 structure (AlphaFold) [36] as well as the DynaMut [37] predictions of protein stability change upon the detected amino acid substitutions can be found in Supplemental Figs. 4–6 and Supplemental Table 3.

Phenotypes are listed in Table 2 and compare with previously-reported cases [10, 11, 21–24]. All had multi-drug resistant focal segmental glomerulosclerosis (FSGS), progressing rapidly to CKD stage 5 within 2–12 months and required kidney transplant. Atypically for monogenic SRNS, post-transplant disease recurrence occurred within 24 h in 1/5 cases, S013682, responding to plasmapheresis (PE) daily for 2 weeks then × 2 weekly for 4 weeks. At 13 years post-transplant, graft function is good with no evidence of proteinuria. Patient 7 passed away 2 years after the transplant from respiratory failure. Four out of 5 patients had microscopic haematuria pre-transplantation as described previously in SRNS caused by *NUP93* mutations [11].

### Histology

**Fig. 2:**
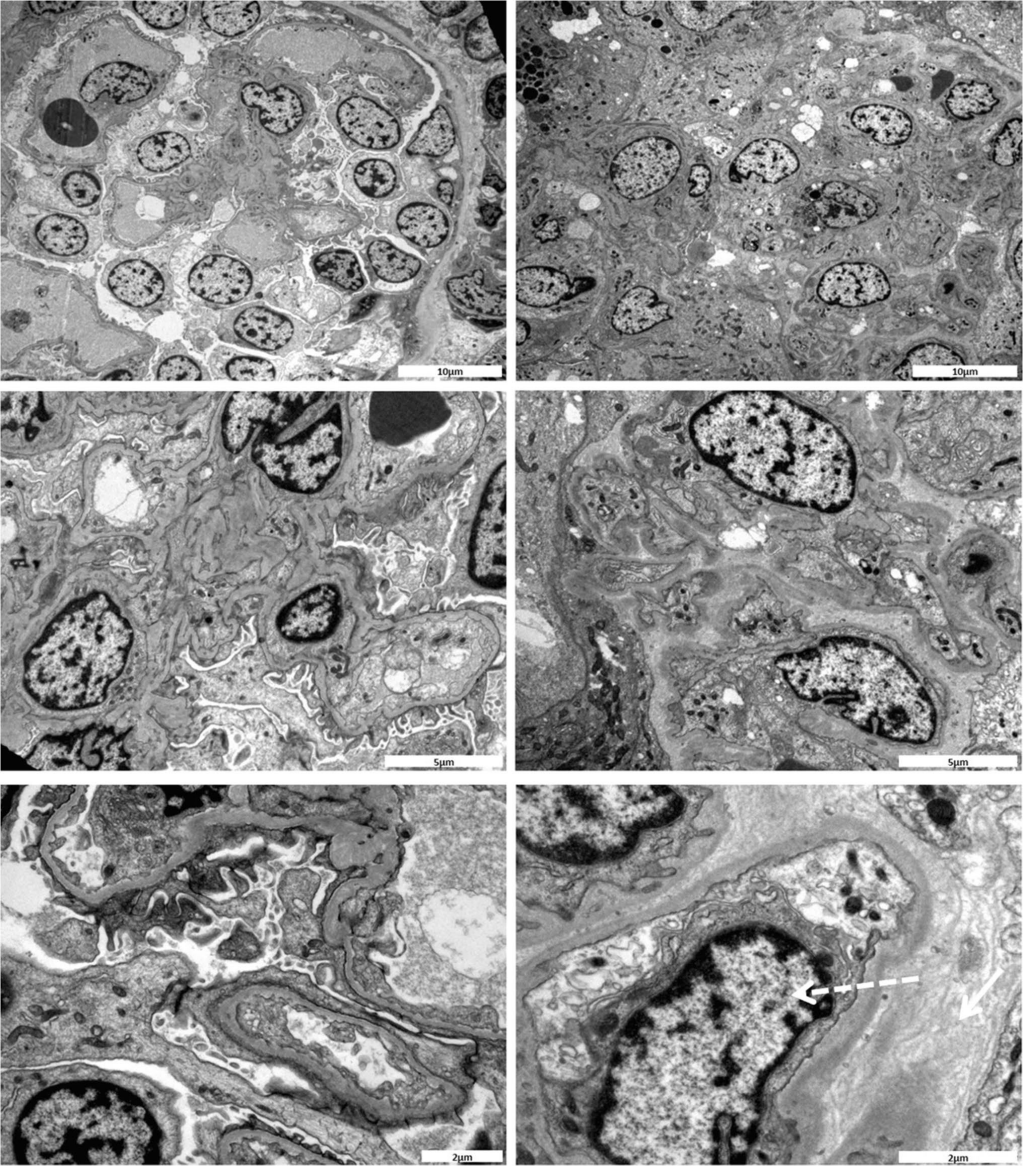
Electron microscopy images of a kidney biopsy sample from patient 7 and 7S**.** Left panel shows electron micrographs of kidney biopsy sample from patient 7. There is gross foot process fusion with only a few areas showing intact foot processes. Otherwise, the glomeruli are of normal architecture without evidence of electron dense deposit. There is no significant mesangial thickening and GBMs appear of normal thickness. Right panel shows electron micrographs of kidney biopsy sample from patient 7S. The glomerulus is partly contracted. Endothelial hyperplasia and subendothelial oedema are observed (dashed arrow). In some areas of the GBM there appears to be some lamination and thickening of the basement membrane together with a basketweave-like appearance that is on the epithelial (outside) aspect of the membrane (solid arrow)

All 5 patients had FSGS on biopsy. Patient 7 (Fig. 2) also had features of collapsing glomerulopathy, and together with his sibling 7S, tubular dilatation. Patients 7S, 85, S013682 and S013282 had interstitial fibrosis and tubular atrophy consistent with previous reports of *NUP93* patients [10, 11]. Full histological description is provided in Supplementary Data.

### *D. melanogaster* model

In view of these findings, and the similarities between human podocytes and *D.melanogaster* nephrocytes, we developed an animal model to investigate the effect of *Nup93* knockdown.

**Fig. 3:**
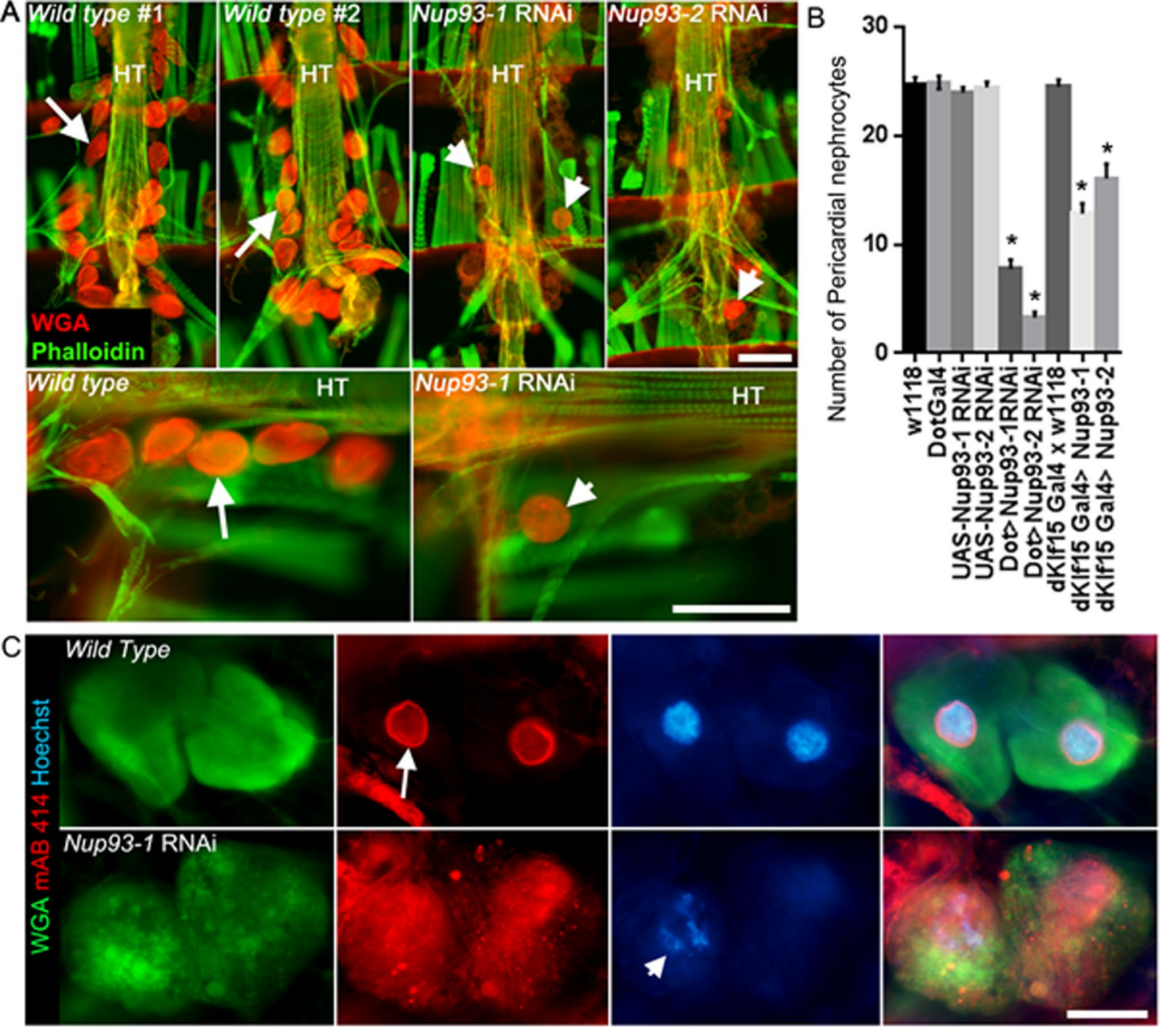
*NUP93* is crucial for Drosophila pericardial nephrocyte development. (**A**) Micrographs of the adult Drosophila heart stained with wheat germ agglutinin (WGA, red) and phalloidin (green). The heart tube (HT) of wild-type flies (2 individuals are shown) is flanked by kidney-like nephrocytes (arrows) which preferentially bind WGA. In contrast, fewer nephrocytes are seen in adults when the expression of either *NUP93-1* or *NUP93-2* is silenced by RNAi using a nephrocyte-restricted driver Dot-Gal4 (arrowheads). Scale bars = 100 µm. 7. **(B)** Quantified data indicate a significant reduction in nephrocyte numbers in flies where *NUP93-1* or *NUP93-2* were silenced using either DotGal4 or dKlf15Gal4 drivers, compared to wild type (w1118) or parent lines (DotGal4; dKlf15Gal4; UAS-*NUP93-1* RNAi or UAS-*NUP92-2* RNAi). *n* = 22–35 different flies for each genotype. *P < 0.001 compared to w1118 control line. **(C)** Micrographs show nephrocytes stained with anti-nucleopore protein antibody (mAB 414, red), wheat germ agglutinin (WGA, green; cell membranes) and Hoechst (blue, nucleic acids). Nephrocytes in wild-type flies (in this instance, the driver line outcrossed to w1118) show distinct perinuclear staining of nucleopore proteins (arrow), whereas the nucleus and perinuclear staining for nucleopore proteins is severely disrupted in nephrocytes when *NUP93-1* was silenced (arrowhead, dKlf15Gal4 > NUP93-1 RNAi). Scale bar = 20 µm

The impact of silencing *Nup93* in nephrocytes is shown in Fig. 3. Silencing *Nup93-1* or *Nup93-2* using nephrocyte-restricted drivers led to a significant reduction in adult pericardial nephrocyte numbers (Fig. 3A and B). Using the *DotGal4* driver, nephrocyte numbers were reduced by 70% (*Nup93-1* silencing; *P* < 0.001) or 82% (*Nup93-2* silencing; *P* < 0.001). Similarly, using the *dKlf15Gal4* driver, nephrocyte numbers were reduced 50% and 36% (*Nup93-1* and *Nup93-2*, respectively; *P* < 0.001 for both compared to controls). Antibody staining against the nuclear pore complex proteins was robust in wild type and parent lines but grossly disrupted, with loss of nuclear membrane localisation, in *Nup93-1* or *Nup93-2* silenced nephrocytes. In addition, nephrocytes in flies surviving to adulthood showed enlarged and granular morphology in *Nup93-1* and *Nup93-2* silenced flies (Fig. 3C).

**Fig. 4:**
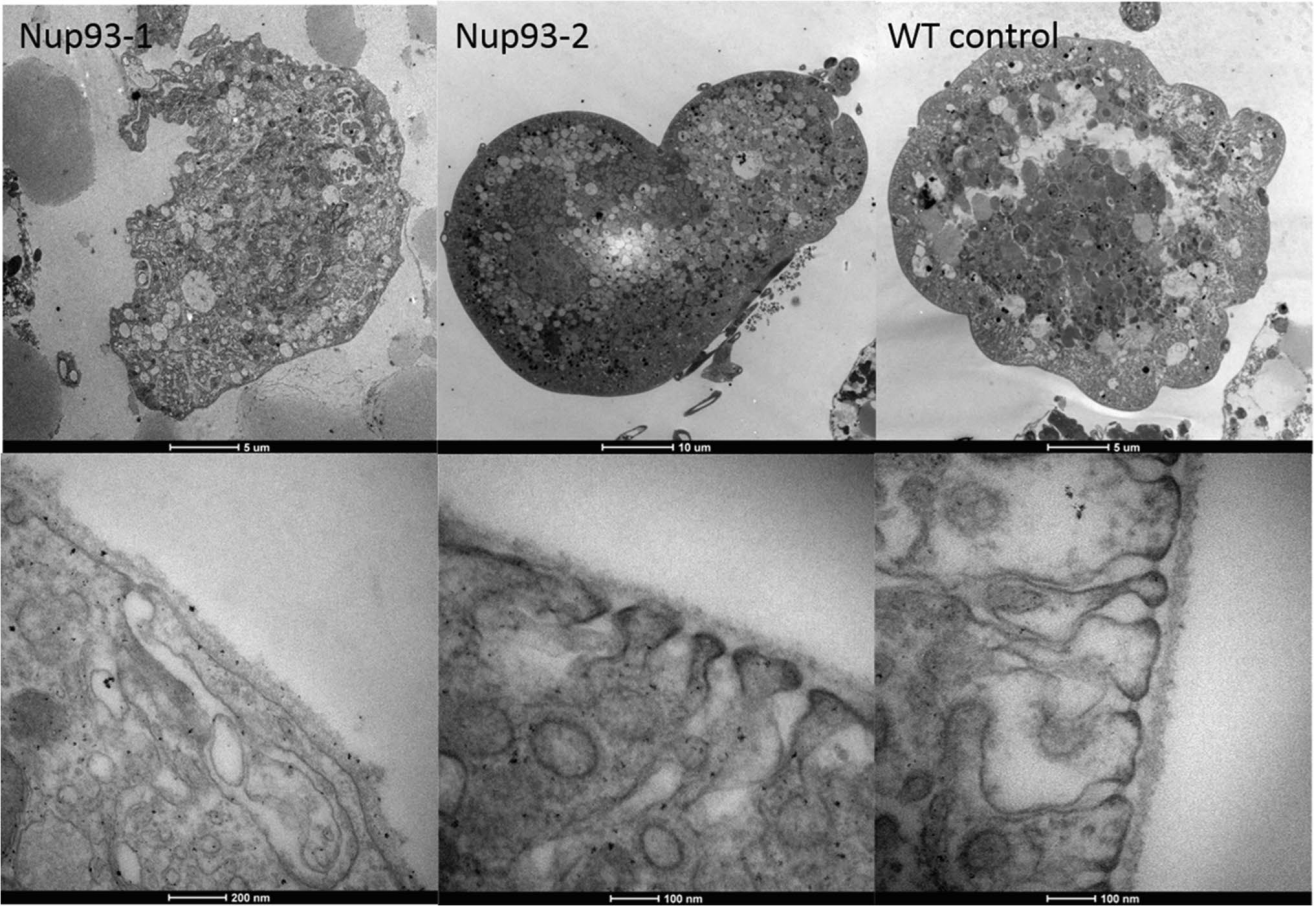
Electron microscopy of *NUP93* mutants and wild type Drosophila nephrocytes. Top panel shows low power sections, bottom panel shows higher power sections. ***NUP93-1***: A collapsed cellular profile with indented margins and large vacuoles abundant in the cytoplasm. Basement membrane is intact with very few fenestrations leading into large interconnecting sub-membranous spaces containing membrane bound inclusions. ***NUP93-2****:* An over-inflated cellular profile with large vacuoles abundant in the cytoplasm. Basement membrane is intact with fewer fenestrations than control leading into a well-defined narrow sub-membranous labyrinth. **Wild type control**: An inflated cellular profile with large vacuoles abundant in the cytoplasm. Basement membrane is intact with abundant fenestrations leading into well-defined narrow sub-membranous labyrinths

Wild type nephrocytes were inflated with a convex surface on EM, with slit-like structures highly similar to the podocyte filtration system [13] (Fig. 4). The cytoplasmic side of these openings into the cell interior are dense where they approximate to the external membrane. These slits lead into a sub-membranous labyrinth of interconnected spaces with an external well-formed basement membrane (Fig. 4, WT control higher magnification).

Mutant nephrocytes had an increased number of dents or concave surface regions at low magnification (Fig. 4*Nup93-1*). At times, cells were swollen (Fig. 4*Nup93-2*) with the cell surface only partially covered in slits leading to a disrupted sub-membranous labyrinth (Fig. 4*NUP93-1* and *NUP93-2* higher magnification). The external basement membrane appears similar to the control.

## Discussion

To date, mutations in 7 nuclear pore genes, *NUP85, NUP107, NUP133, NUP160, NUP205, XP05*, and *NUP93* have been implicated in SRNS [11, 19, 20], with 16 patients (15 families) described with *NUP93*. Here, we report a further 6 novel and previously undescribed *NUP93* variants in 3 sporadic (85, S013282 and S013682) and 2 familial (7 and 7S) paediatric-onset SRNS cases. Of note, the most common previously-identified p.(Gly591Val) *NUP93* mutation (9/16 cases) was absent in our cohort suggestive of a founder effect in those patients [11, 21]. We used stringent phenotyping as well as bioinformatic approaches for genetic data analysis and also used these to support in silico the possibly pathogenic role in the detected variants (variants were considered as possibly disease-causing when they were novel or very rare and predicted to affect protein [25]). While we provide compelling computational evidence for their potential pathogenicity and the fact that they are likely causal in at least 4 of these individuals’ disease (especially in the absence of any other candidate genes), each variant would require further functional confirmation either in a cellular or animal model or identified in other unrelated SRNS patients.

However, *NUP93* mutations have only recently been associated with SRNS, and as such only a small number of pathogenic mutations in limited kindreds have been identified to date. As a result, and in common with other new genes in rare diseases, mutational hotspots or critical regions have yet to be identified. This lack of gene data and absence of functional proof in a cellular or animal model meant that despite strong computational evidence for likely pathogenicity and robust genotype–phenotype correlation, for novel or very rare variants current ACM guidelines are often not met [38]. Consequently, without functional assay and/or identification in other (unrelated) SRNS patients, not only the *NUP93* variants described in this paper but the majority previously published, would be classified as variants of unknown significance (VUS). This leads to the important discussion about how best to apply guidelines in rare disease bearing in mind the rapid and increasing application of next generation sequencing as a diagnostic tool in kidney medicine. Fortunately, interpretation using a composite picture made up of phenotype, genotype and results of conventional tests such as kidney biopsy can refine the likelihood of a lab report of a “VUS” as more likely pathogenic, pending results of functional assay or more gene data.

Our data is consistent with previous reports [10, 11, 21–24, 39, 40], supporting *NUP93* mutations as a cause of paediatric onset SRNS. Familial cases had homozygous changes p.(Ala475Thr, 7 and 7 s) and p.(Lys637Glu, S013682) and were diagnosed in the first 2 years of life with rapid onset kidney failure. Sporadic patients 85 and S013282 were diagnosed at age 6 and 8 respectively with later-onset SRNS but correspondingly dramatic decline in eGFR within 2–12 months. All 5 had FSGS, tubular atrophy and dilatation, hyaline casts on kidney biopsy and haematuria consistent with previous reports [10, 11]. Unusually for multidrug resistant monogenic disease [41], S013682 developed post-transplant disease recurrence responding to plasma exchange (PEX). Interestingly, post-transplant disease recurrence responding to PEX has been described previously in a case of SRNS caused by compound heterozygous *NUP93* mutation (p.Gly591Val + p.Leu639Pro) [21, 22]. In contrast to S013682 successfully treated with PEX alone, the case became PEX dependent and required rituximab [22]. Post-transplant recurrence is rare in monogenic SRNS and is generally mediated by antibodies, most descriptions being of anti-nephrin antibodies in Finnish type congenital nephrotic syndrome [42]. Equally, there are links between nucleoporins and innate immunity raising the possibility of an as yet unidentified mechanism. The other possibility is that the *NUP93* variant in this patient is benign, emphasising the need for improved prediction models.

To this end we confirm that nephrocyte-specific knockdown of *Nup93* in vivo is deleterious, supporting a direct effect of *NUP93* loss of function mutations on podocyte function and therefore a pathogenic role in SRNS. Since novel mutations without supporting functional data tend to be classified as “VUS”, our Drosophila in vivo model could be modified to assay *NUP93* mutations to determine pathogenicity in this and other SRNS cohorts.

The need for better experimental models is illustrated well by our patient with post-transplant recurrence. The location of p.(Lys637Glu) is within a region described for other *NUP93* mutations. In addition, this novel variant is absent in healthy ethnically-matched controls and commonly used bioinformatics analysis determined a deleterious score for this amino acid substitution. However, the evolutionary model of variant effect (EVE) recently published in *Nature* [43], predicts the amino acid substitution to be benign. Alleles were inherited in trans, i.e., one from each unaffected heterozygous parent. Homozygosity mapping analysis further supported the variant being the strongest candidate in this patient. Functional work such as in our fly model, would be required at this stage to confirm pathogenicity and reclassify this variant from “uncertain significance.” Post-transplant recurrence observed in patient S013682 remains unexplained and the relationship between the genetic variant and recurrence remains to be seen. However, this is the second case of post-transplant disease recurrence associated with a *NUP93* mutation. Sandokji et al. recently reported a 5-year-old girl presenting with SRNS, cardiomyopathy and developmental delay with autistic features. After kidney transplant, she had self-limiting proteinuria which resolved within a week [24]

Nucleoporins are known to have a role in the immune system. Although there is no corresponding *NUP93* mouse model, *NUP210* knockout mice develop peripheral T-cell alterations [44] while *NUP96* + / − animals present selective alterations of the immune system [45]. Furthermore, NUP93 interacts with SMAD4, a signaling protein, and *NUP93* mutations have been shown to abrogate SMAD activity [11]. SMAD4 has a critical role in T-cell function and is required in T-cell-mediated autoimmunity and tumour rejection [46]. NUP93 is also known to regulate antiviral innate immune responses [47] and is expressed in peripheral blood mononuclear cells as well as in the kidney [10]. This apparent link between the immune system and other nucleoporins, together with NUP93 expression in mononuclear cells, supports a relationship with immune function as does the detection of anti-NUP210 and -NUP62 antibodies in primary biliary cirrhosis, systemic lupus erythematosus, autoimmune myositis and rheumatic diseases [48]. Nevertheless, a link with risk of disease recurrence post-transplant remains unproven.

In common with other nucleoporins [20] the *NUP93* phenotype can either be a kidney-specific disease or syndromic with neurodevelopmental features and cardiac anomalies [24]. NUP93 is highly expressed in the human brain and cerebellum and *NUP93* mutations were recently linked to autosomal recessive congenital ataxia [49]. While none of our cases had ataxia, autistic spectrum disorder was present in 1 of our patients, S013282, and more non-specific features of neurodevelopmental delay in the other patients as might be expected. This confirms the previously noted association and affirms that SRNS cases with *NUP93* mutations may also have neurological involvement.

NUP93 is expressed in all human kidney cell types [10]. Cellular knockdown of *NUP93* in vitro has been shown to be significantly important in cell functions such as nuclear pore assembly, cell migration, and interaction with signaling cascading mediated by other NUP’s, SMAD4 and importin 7 [11, 50]. Since *D. melanogaster* and humans share conserved roles in a number of cellular processes, we opted to use the fly’s equivalent of podocytes, the nephrocyte, to investigate a role for *NUP93* in SRNS.

We created the first in vivo nephrocyte specific knockdown of *Nup93*. Examination of our experimental model indicated that nephrocyte development requires the expression of both human *NUP93* orthologues, ubiquitously expressed *Nup93-1* and nephrocyte-specific *Nup93-2*. Silencing expression using either of 2 well-defined, nephrocyte-restricted drivers led to a reduced number of nephrocytes with surviving cells demonstrating highly abnormal morphology. Neither the *Nup93-1* nor the *Nup93-2* RNAi lines are predicted to have off-target effects, and silencing of either independently caused a severe phenotype, indicating a lack of functional compensation. These findings support using a fly model as a functional assay to determine the pathogenicity of mutations detected in human disease.

In summary, we detected 6 new *NUP93* variants in autosomal recessive paediatric-onset SRNS expanding the variation spectrum and confirming that *NUP93* is associated with a phenotype of multi-drug resistant, rapidly progressive SRNS. Phenotypic expansion to include neurodevelopmental features if syndromic disease is present is also confirmed. The potential for *NUP93* mutations as causal in podocytopathy is supported mechanistically with the finding that silencing either *Nup93-1* or *Nup93-2* leads to a severe and non-redundant disruption of *D. melanogaster* nephrocyte development and function. Our data underpins the difficulties in confirming mutations as pathogenic in rare diseases with recently described gene associations and with only a few cases described, despite strong correlation between phenotype and genotype. While it is recognised that this is difficult to implement in the clinic, the rapid generation of data using next generation sequencing and the current difficulties in interpreting “pertinent” from “incidental” supports the need for functional assay of mutations such as in the fly kidney model presented here.

## Supplementary Information

Below is the link to the electronic supplementary material.

Supplementary file1 (DOCX 3058 KB)

Supplementary file2 (PPTX 1.14 MB)

## Data Availability

All data and materials are stored according to Ethical approvals at Bristol Renal laboratories.
