# Supplementary material for: Exploring the relevance of NUP93 variants in steroid-resistant nephrotic syndrome using next generation sequencing and a fly kidney model
Source: Pediatr Nephrol. 2022 Feb 24;37(11):2643–56. doi: 10.1007/s00467-022-05440-5 (PMC9489583; doi:10.1007/s00467-022-05440-5)
Supplement: Supplementary file 1 — Supplementary file1 (DOCX 3058 KB) [file 467_2022_5440_MOESM1_ESM.docx]

**Supplementary data**

**Table 1 Genes associated with nephrotic syndrome [1-15]**

FSGS, Focal segmental glomerulosclerosis; CNS, congenital nephrotic syndrome; NS, nephrotic syndrome; SRNS, Steroid resistant nephrotic syndrome; SSNS, Steroid Sensitive Nephrotic Syndrome; SDNS, steroid dependent NS; DMS, diffuse mesangial sclerosis; AD, autosomal dominant; AR, autosomal recessive, DMS, Diffuse mesangial sclerosis; MPGN, Membranoproliferative glomerulonephritis; CKD, chronic kidney disease

| **Gene** | **Inheritance** | **Disease** |
| --- | --- | --- |
| *ACTN4* | AD | Familial and sporadic SRNS (usually adult) |
| *ADCK4* | AR | SRNS |
| *ALG1* | AR | Congenital disorder of glycosylation |
| *ANKFY1* | AR | paediatric SRNS |
| *ANLN* | AD | FSGS (mainly adult) |
| *ARHGAP24* | AD | FSGS |
| *ARHGDIA* | AR | Congenital nephrotic syndrome |
| *AVIL* | AR | SRNS |
| *CD151* | AR | NS, pretibial bullous skin lesions, neurosensory deafness, bilateral lacrimal duct stenosis, nail dystrophy, and -thalassemia minor |
| *CD2AP* | AD/AR | FSGS/SRNS |
| *CFH* | AR | MPGN type II + NS |
| *CLCN5* | X-linked recessive | Dent’s disease +/- FSGS +/- hypercalcuria and nepthrolithiasis |
| *COL4A3* | AR | Alport’s disease/FSGS |
| *COL4A4* | AR | Alport’s disease/FSGS |
| *COL4A5* | X-linked recessive | Alport’s disease/FSGS |
| *COQ2* | AR | Mitochondrial disease/isolated nephropathy |
| *COQ6* | AR | NS +/- sensorineural deafness; DMS |
| *CRB2* | AR | SRNS |
| *CUBN* | AR | Intermittent nephrotic range proteinuria +/- with epilepsy |
| *DGKE* | AR | Haemolytic-Uremic Syndrome + SRNS |
| *DLC1* | AR | childhood and adult SSNS and SRNS |
| *E2F3* | AD | FSGS+mental retardation (whole gene deletion) |
| *EMP2* | AR | Childhood-onset SRNS and SSNS |
| *FAT1* | AR | Combination of SRNS, tubular ectasia, haematuria and facultative neurological involvement |
| *GAPVD1* | AR | early-onset NS |
| *INF2* | AD | Familial and sporadic SRNS, FSGS-associated Charcot-Marie-Tooth neuropathy |
| *ITGA3* | AR | Congenital interstitial lung disease, nephrotic syndrome, and mild epidermolysis bullosa |
| *ITGB4* | AR | epidermolysis bullosa and pyloric atresia + FSGS |
| *ITSN1* | AR | CNS/SRNS/SSNS (with MCD/FSGS on biopsy) |
| *ITSN2* | AR | SSNS/SDNS (with MCD/MPGN on biopsy) |
| *KANK1* | AR | SSNS |
| *KANK2* | AR | SSNS/SDNS +/- haematuria |
| *KANK4* | AR | SRNS + haematuria |
| *LAGE3* | AR | NS with primary microcephaly |
| *LAMA5* | AR | childhood NS |
| *LAMB2* | AR | Pierson syndrome |
| *LMNA* | AD | Familial partial lipodystrophy + FSGS |
| *LMX1B* | AD | Nail patella syndrome/FSGS without extrarenal involvement |
| *MAFB* | AD | FSGS with Duane Retraction Syndrome |
| *MAGI2* | AR | NS +/- neurological impairment |
| *MYO1E* | AR | Familial SRNS |
| *NEU1* | AR | nephrosialidosis (Sialidosis Type II+childhood NS) |
| *NPHP4* | AR | nephronophthisis with FSGS and nephrotic range proteinuria |
| *NPHS1* | AR | Congenital nephrotic syndrome/SRNS |
| *NPHS2* | AR | CNS, SRNS |
| *NUP107* | AR | Childhood SRNS |
| *NUP133* | AR | SRNS |
| *NUP160* | AR | SRNS |
| *NUP205* | AR | Childhood SRNS |
| *NUP85* | AR | SRNS |
| *NUP93* | AR | Childhood SRNS |
| *NXF5* | X-linked recessive | FSGS with co-segregating heart block disorder |
| *OCRL* | X-linked recessive | Dent disease2, Lowe syndrome, +/- FSGS, +/- nephrotic range proteinuria |
| *OSGEP* | AR | NS with primary microcephaly |
| *PAX2* | AD | adult onset FSGS without extrarenal manifestations |
| *PDSS2* | AR | Leigh syndrome |
| *PLCe1* | AR | Congenital nephrotic syndrome/SRNS |
| *PMM2* | AR | Congenital disorder of glycosylation |
| *PODXL* | AD | FSGS |
| *PTPRO* | AR | NS |
| *SCARB2* | AR | Action myoclonus renal failure syndrome +/-hearing loss |
| *SGPL1* | AR | primary adrenal insufficiency and SRNS |
| *SMARCAL1* | AR | Schimke immuno-osseous dysplasia |
| *SYNPO* | AD | sporadic FSGS (promoter mutations) |
| *TBC1D8B* | X-linked | early-onset SRNS with FSGS |
| *TNS2* | AR | SSNS/SDNS (with MCD/FSGS/DMS on biopsy) |
| *TP53RK* | AR | NS with primary microcephaly |
| *TPRKB* | AR | NS with primary microcephaly |
| *TRPC6* | AD | Familial and sporadic SRNS (mainly adult) |
| *TTC21B* | AR | FSGS with tubulointerstitial involvement |
| *WDR73* | AR | Galloway-Mowat syndrome (microcephaly and SRNS) |
| *WT1* | AD | Sporadic SRNS (children—may be associated with abnormal genitalia); Denys-Drash and Frasier syndrome |
| *XPO5* | AR | Childhood SRNS |
| *ZMPSTE24* | AR | Mandibuloacral dysplasia with FSGS |
| *MYH9* | AD/assoc. | MYH9-related disease; Epstein and Fechtner syndromes |
| *APOL1* | G1, G2 risk alleles (AR) | Increased susceptibility to FSGS and CKD in African Americans, Hispanic Americans and in individuals of African descent |

**Figure 1** **NUP93 c.1538-6A>G mutation found in patient S013282**

A screen shot from Alamut Visual (Alamut Visual version 2.15 (SOPHiA GENETICS, Lausanne, Switzerland) showing splicing predictions for the NUP93 c.1538-6A>G heterozygous variant found in patient S013282. The top panel shows reference sequence and the bottom panel sequence containing the NUP93 variant


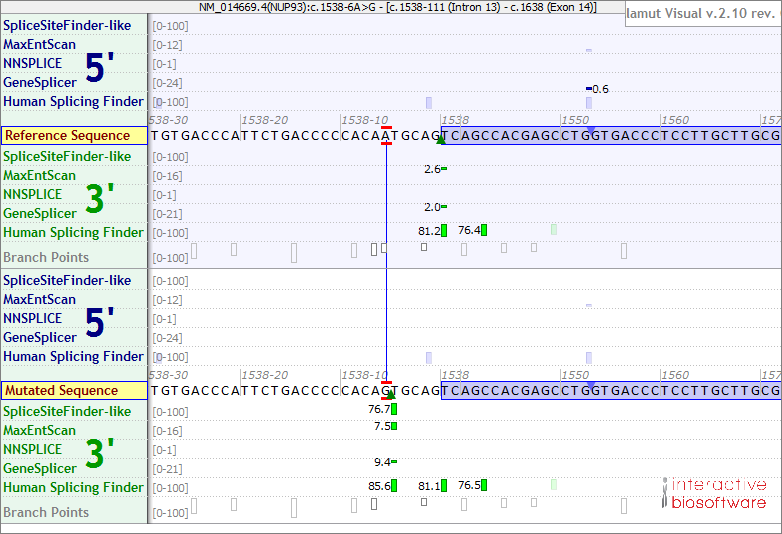


**Figure 2 NUP93 c.1473T>G:** **p.His491Gln mutation found in patient S013282**

A screen shot from Alamut Visual (Alamut Visual version 2.15 (SOPHiA GENETICS, Lausanne, Switzerland) showing splicing predictions for the NUP93 c.1909A>G; p.(Lys637Glu) heterozygous variant found in patient S013682. The top panel shows reference sequence and the bottom panel sequence containing the NUP93 variant


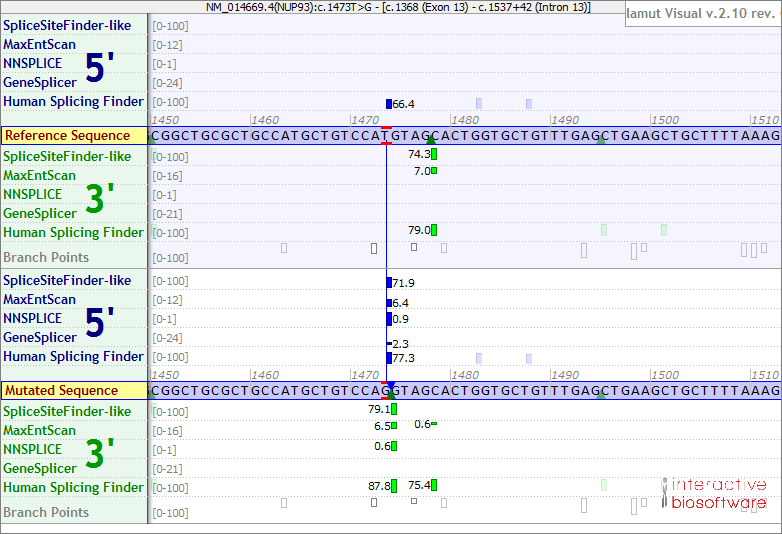


**Figure 3 Homozygosity plot - S013682**

Homozygosity mapping (<http://www.homozygositymapper.org/>)[16] was performed for individual S013682 since the parents are consanguineous and to see whether there is any better mutation candidate than the NUP93 variant in this patient. Homozygosity plot is shown with chromosome numbers presented on x-axis and score on y-axis. Red bars represent regions of homozygosity, which reach maximum significance (height of red bar) on all chromosomes apart from 7, 14, 15, and 20. Only 4 homozygous variants with MAF < 0.01 were found within the homozygosity regions that are not present in gnomAD or in in-house controls in a homozygous state and are affecting a conserved amino acid. One of the variants is the NUP93 p.(Lys637Glu). The other 3 variants are in NR1I2, IER5L and LAMC3 genes which are not linked to a kidney phenotype.


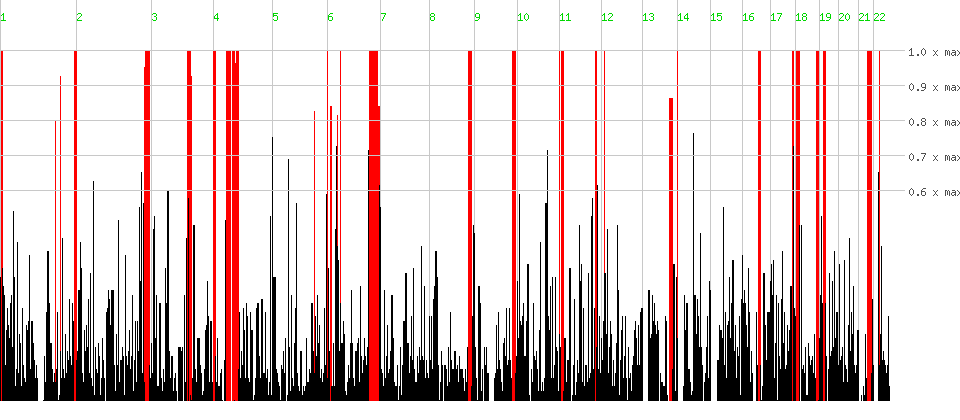


**Table 2 Rare homozygous variants found within homozygosity runs in patient S013682**

Homozygous variants found within homozygosity runs in the coding sequence of patient S013682 were filtered. Variants with frequency (MAF) > 0.01, found as homozygotes (gnomAD v.2.1.1 or our in-house control database) or affecting a not conserved amino acid (UCSC Genome Browser) were filtered out. Four variants remained. Only *NUP93* gene is known to be associated with a kidney phenotype. gnomAD v2.1.1 (The Genome Aggregation Database, <http://gnomad.broadinstitute.org/>

AF, allele frequency; AC, allele count; AH, number of homozygotes; AN, allele number

In sillico predictions (SIFT, PolyPhen, MutationTaster, REVEL and CADD Phred scores) were obtained from Ensembl Variant Effect Predictor (VEP, <https://www.ensembl.org/info/docs/tools/vep/index.html>)

* variants with low scores for being likely deleterious. Known disease associations for each gene were obtained from GeneCards (https://www.genecards.org/)

| **Gene** | **Variant** | **gnomAD 2.1.1 AF (AC/NH/AN)** | **SIFT** | **PolyPhen** | **MutationTaster** | **REVEL** | **CADD** | **Disease associations (GeneCards)** |
| --- | --- | --- | --- | --- | --- | --- | --- | --- |
| NUP93 | c.1909A>G: p.Lys637Glu | . | 0.12* | 0.56 | 1 | 0.372* | 24.8 | Nephrotic Syndrome |
| NR1I2 | c.1109C>A: p.Ala370Asp | . | 0 | 0.909 | 0.92 | 0.63 | 23 | Cerebrotendinous Xanthomatosis and Biliary Tract Disease |
| IER5L | c.349C>G: p.Leu117Val | . | 0.03 | 0.579 | 0.65 | 0.132* | 24.2 | Spinal Muscular Atrophy, Type Iv and Retinitis Pigmentosa 7 |
| LAMC3 | c.1672C>T: p.Leu558Phe | 0.00002387 (6/0/251346) | 0 | 0.922 | 0.94 | 0.333* | 22.7 | Cortical Malformations, Occipital and Tooth Resorption |

**Table 3 DynaMut *NUP93* variant predictions based on the AlphaFold 3D human *NUP93* structure**

DynaMut prediction results of the detected *NUP93* amino acid substitutions and the ΔΔG predictions for well-established methods that are based on protein structure using different approaches and assumptions (machine learning, NMA, substitution tables) for comparison purposes [17]. AlphaFold 3D human *NUP93* structure (AF-Q8N1F7-F1)[18] was used for the predictions.

| **Amino acid substitution** | **Human NUP93** | **Ala475Thr** | **His491Gln** | **Lys637Glu** | **Leu695Ser** | **Leu756Ser** |
| --- | --- | --- | --- | --- | --- | --- |
| **DynaMut Prediction Outcome** | **ΔΔG [kcal/mol]** | -0.201 (Destabilizing) | 0.235 (Stabilizing) | -0.207 (Destabilizing) | -3.823 (Destabilizing) | -3.638 (Destabilizing) |
| **NMA Based Predictions** | **ΔΔG ENCoM [kcal/mol]** | 0.423 (Destabilizing) | -0.205 (Destabilizing) | -0.178 (Destabilizing) | -0.617 (Destabilizing) | -0.561 (Destabilizing) |
| **Other Structure-Based Predictions** | **ΔΔG mCSM [kcal/mol]** | -1.030 (Destabilizing) | -2.119 (Destabilizing) | -0.741 (Destabilizing) | -3.449 (Destabilizing) | -3.352 (Destabilizing) |
|  | **ΔΔG SDM [kcal/mol]** | -3.320 (Destabilizing) | -1.230 (Destabilizing) | 0.650 (Stabilizing) | -4.190 (Destabilizing) | -4.310 (Destabilizing) |
|  | **ΔΔG DUET [kcal/mol]** | -1.307 (Destabilizing) | -2.150 (Destabilizing) | -0.209 (Destabilizing) | -3.776 (Destabilizing) | -3.695 (Destabilizing) |

**Figure 4 Location of the detected *NUP93* variants on the AlphaFold 3D structure prediction of human *NUP93***

**A**. Conservation across evolution of altered amino acids for the 5 missense variants Alamut Visual Pro (SOPHiA GENETICS, Lausanne, Switzerland **B**. AlphaFold 3D predicted human *NUP93* structure (AF-Q8N1F7--F1) [18] with affected amino acids indicated by red arrows. Model Confidence: navy blue - Very high (pLDDT > 90), light blue - Confident (90 > pLDDT > 70), Low (70 > pLDDT > 50), yellow - Very low (pLDDT < 50) [17]


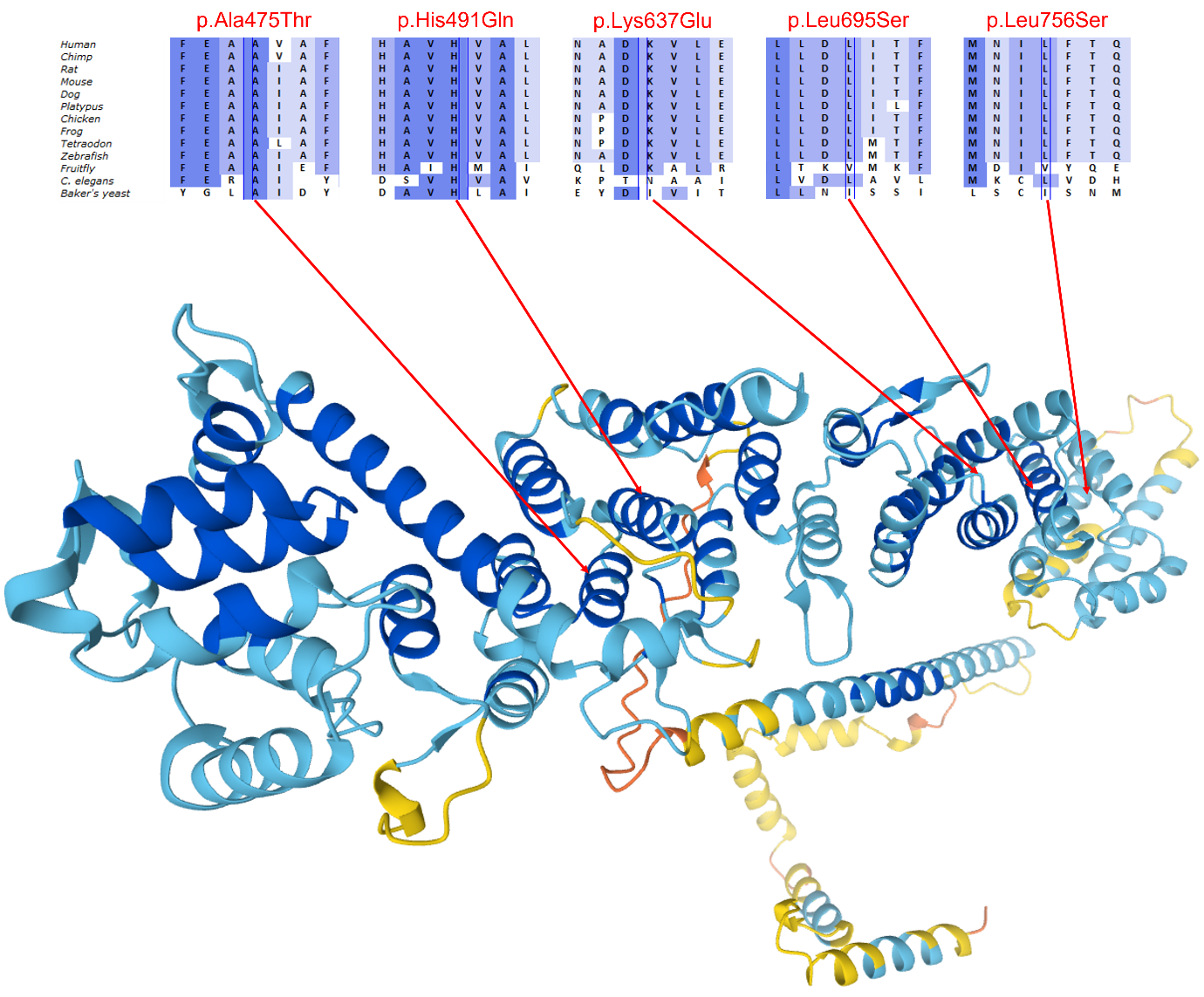


**Figure 5 DynaMut Prediction of Interactomic Interactions of the detected *NUP93* amino acid substitutions**

Wild-type and mutant residues are coloured in light green and are also represented as sticks alongside the surrounding residues which are involved in any type of interactions [17]. AlphaFold 3D human *NUP93* structure (AF-Q8N1F7-F1)[18] was used for the predictions.

*
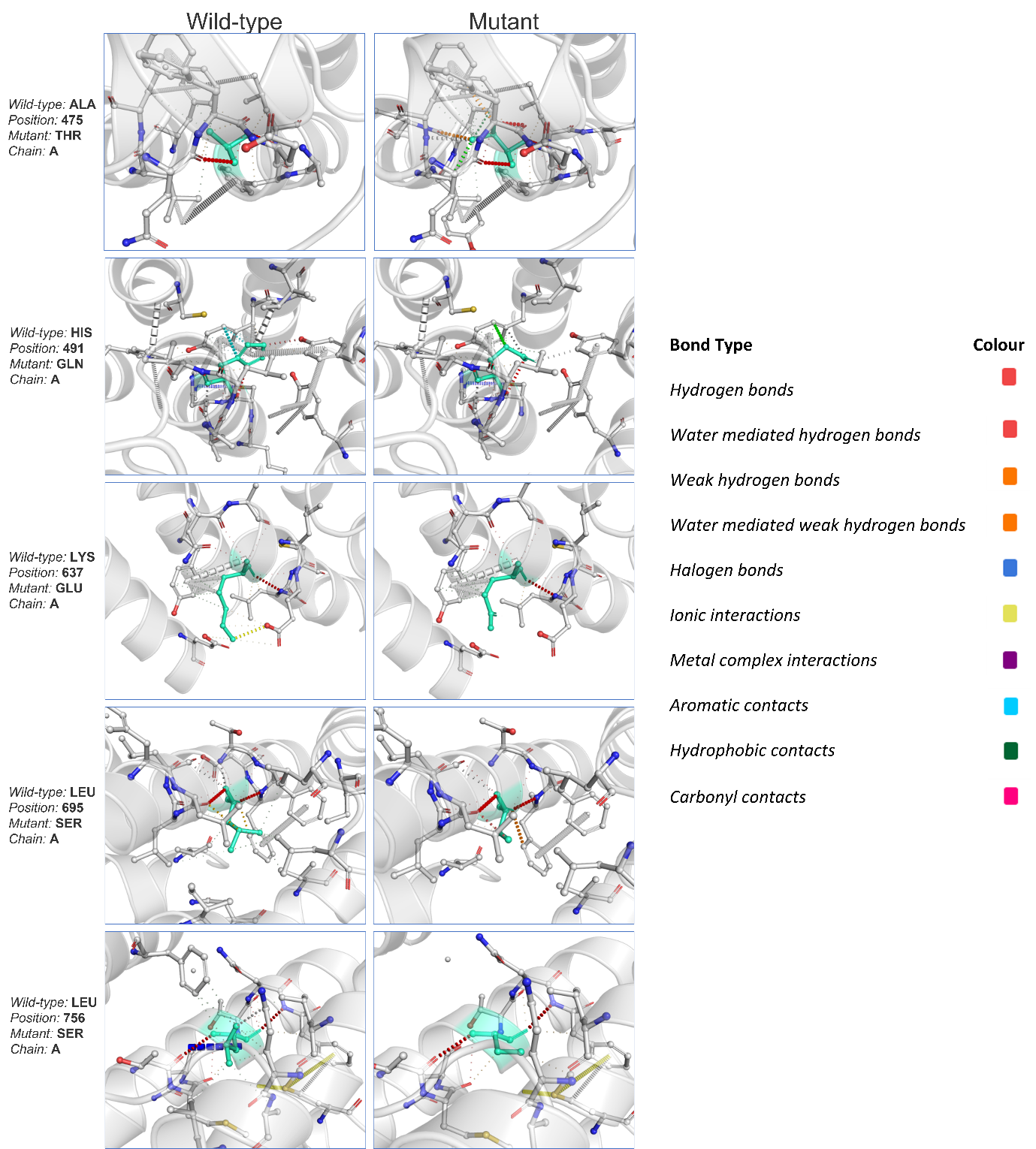
*

**Figure 6 Δ Vibrational Entropy Energy Between *NUP93* Wild-Type and Mutant**

Amino acids coloured according to the vibrational entropy change upon mutation. BLUE represents a rigidification of the structure and RED a gain in flexibility. Predictions obtained using the AlphaFold 3D human *NUP93* structure (AF-Q8N1F7-F1)[18] and DynaMut [17]


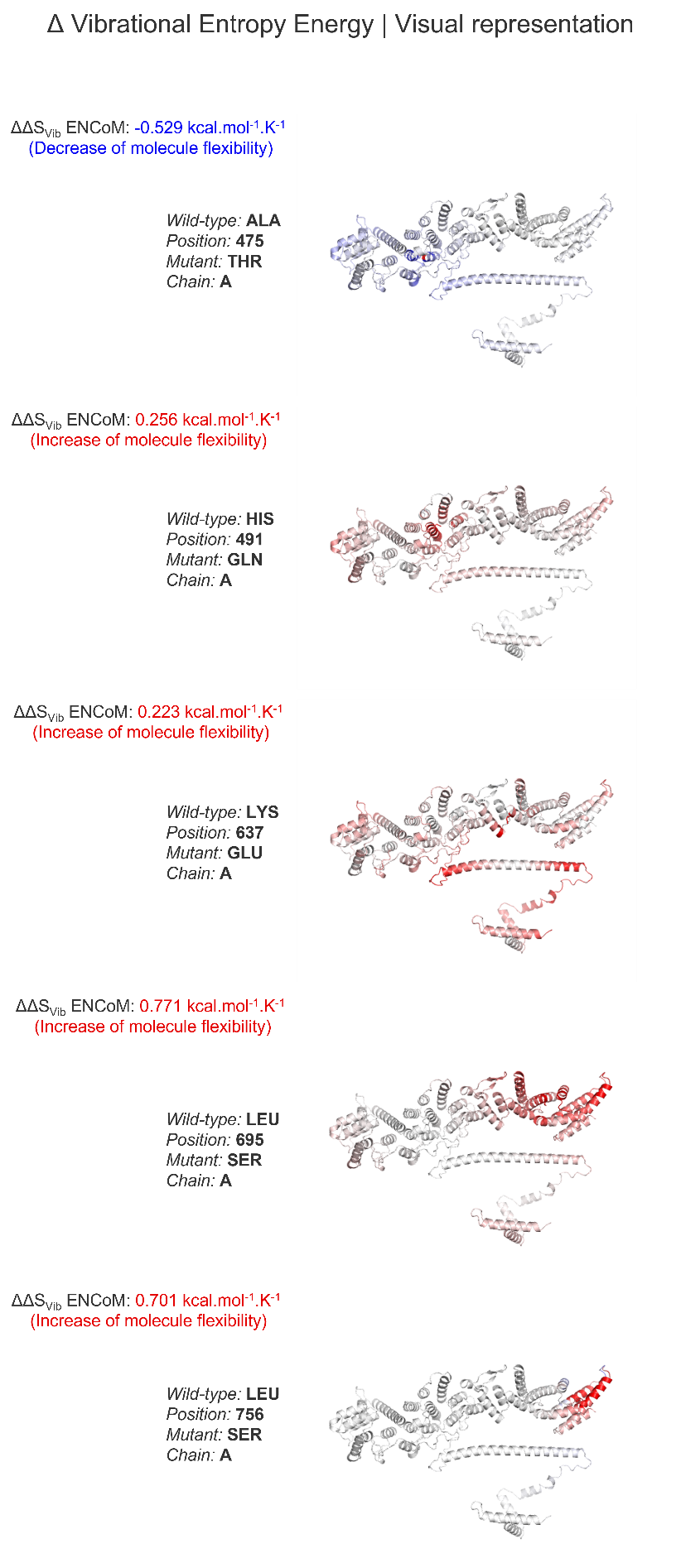


**Variant filtering criteria used**

Stringent rules were used to filter variants (based on the combination of literature, ACMG, the National test directory list of “green” ‘nephrotic genes’) in order to identify those that are definite/probably/possibly pathogenic. Variant filtering and classification were performed as described previously [1] and the following steps were applied:

1. Only variants below MAF 0.01 were considered (except NPHS2 p.R229Q).
2. Variants seen previously as homozygotes in The Genome Aggregation Database (gnomAD), 1000 Genomes Project, or KCL data set (King’s College London in-house data from 3,000 individuals without a kidney disease) were excluded (unless described previously as a mutation).
3. All remaining variants were cross checked with HGMD Professional 2021.3.
4. Prediction Tools used: Alamut Visual Plus (SOPHiA GENETICS, Lausanne, Switzerland; including amino acid conservation, SIFT, MutationTaster), Ensembl Variant Effect Predictor (VEP; including PolyPhen, REVEL score, CADD PHRED score)[19] and evolutionary model of variant effect (EVE)[20]. Missense variants needed to be predicted by the majority of the tools to be considered as potentially deleterious.
5. Using Alamut Visual Plus (SpliceSiteFinder-like, MaxEntScan, NNSPLICE, GeneSplicer) synonymous and splice site variants were considered where there was a consistent predicted splice effect across the majority of tools.
6. Using UCSC (http://genome.ucsc.edu/) and Alamut Visual Pro amino acid must be conserved and not present in another multicellular organism.
7. For compound heterozygotes, where DNA samples from the parents were available, variants were verified if are in trans. In most cases trios were available for verification of trans status to lend support to the pathogenicity of variants and this is stated. If not, this is also stated, and variants interpreted with caution.
8. Variants were considered as a definite mutation only if they were previously described mutations causing SRNS/related phenotype. Variants were considered as probably disease-causing when:
   1. A novel nonsense or frameshift variant (heterozygous for AD genes and homozygous for AR genes) was identified compared with ethnically-matched control databases
   2. A novel variant affecting the same codon as a previously described mutation
   3. A compound heterozygote where one of the variants is a known mutation and the second predicted to be deleterious if novel for the disease
   4. A known mutation causing e.g., Alport syndrome found in a patient without typical features of the syndrome
9. Variants were considered as possibly disease causing if novel (i.e., absent in ethnically-matched control databases), very rare based on usual metrics for MAF and also predicted to have a deleterious impact on protein function when they were novel or very rare and predicted to affect protein.

Aside from multi-sample calling, all variants were confirmed with Sanger sequencing to confirm authenticity and exclude technical artifact.

**Histology**

Patient 7

Established focal segmental glomerular sclerosis with some features of collapsing glomerulopathy. There is no mesangial thickening or hypercellularity; however, a few show some epithelial cell hyperplasia, resembling a small crescent in places associated with some collapse of an underlying glomerular segment. GBMs appear normal. There is a band of atrophy with tubular dilatation with some associated tubules containing eosinophilic casts. The remaining tubules appear normal without interstitial fibrosis. No significant deposit of IgG, IgA, IgM or C3 was identified. Gross podocyte foot process fusion on electron microscopy with only a few areas showing intact foot processes (main text Figure 1).

Patient 7S

Advanced focal segmental glomerular sclerosis. GBMs show non-specific irregularity. There is extensive tubular atrophy with many dilated (cyst-like) tubules. There is interstitial oedema and a mild chronic inflammatory cell infiltrate. There is some apparent granular C3 and widespread IgM on the contracted basement membranes. IgG and IgA are negative. Electron microscopy shows endothelial hyperplasia and subendothelial oedema (main text Figure 1).

Patient 85

Established focal segmental glomerular sclerosis. There is mild and variably segmental mesangial proliferation. Within virtually all of the glomeruli, there is segmental and multi‑segmental sclerosis, expansion of the mesangial matrix, capping of the segmental sclerosing lesions by swollen podocytes, many of which contain intra‑cytoplasmic protein resorption droplets. There are no crescents. Many of the tubules have hyaline casts and some of the tubules are markedly distended by such casts. There is acute tubular injury with flattening of tubular epithelial cells and protein resorption droplets within their cytoplasm. There is extensive tubular atrophy present within approximately 35‑40% of the tissue. In these areas there is an associated chronic inflammatory cell infiltrate. Interstitial fibrosis is present within approximately 40‑45% of the cortical sample. The ultrastructural appearances are of advanced hyalinosing and sclerosing lesions.

Patient S013682

Focal segmental glomerular sclerosis. Only 2 of the perfused glomerular profiles show entirely open capillary beds with mildly increased mesangial cellularity and matrix. Rest of the glomeruli display diffuse increase in mesangial cellularity and segmental scarring with epithelial capping. Cytoplasmic hyaline droplets in the visceral and parietal epithelial cells are also seen along with hyalinosis. About 60% of cortical tubules are atrophic. Nearly all the preserved cortical tubules are dilated with frequent cytoplasmic protein resorption droplets in epithelial cells. Frequent hyaline casts are seen, and many tubules contain cellular debris. In addition, there are several large foci of tubulitis where polymorphonuclear leukocytes and lymphocytes have infiltrated the tubular epithelium. About 60% of cortical interstitium is focally scarred. There is mild degree of arteriolosclerosis. Thrombi are not seen. There are no vasculitic lesions. Immunohistology shows diffuse mesangial, localisation of granular IgM (++) and C1q (++).

Patient S013282

Focal and Segmental Glomerulosclerosis which has progressed to near end stage disease. The morphological appearances in this biopsy show significant global glomerulosclerosis. The glomeruli that are not completely obsolete show segmental and multisegmental sclerosing lesions. Capillary loops remain patent and perfused where there is not segmental sclerosis. There are no tubulo-reticular inclusions; mesangial cells are normal. There is an increase in mesangial matrix but there are no electron dense deposits. There is diffuse effacement of the foot processes over 80% of the surface area of the glomerular capillary walls. This is associated with microvillous change.

**References**

1. Bierzynska A, McCarthy HJ, Soderquest K, Sen ES, Colby E, Ding WY, Nabhan MM, Kerecuk L, Hegde S, Hughes D, Marks S, Feather S, Jones C, Webb NJ, Ognjanovic M, Christian M, Gilbert RD, Sinha MD, Lord GM, Simpson M, Koziell AB, Welsh GI, Saleem MA (2017) Genomic and clinical profiling of a national nephrotic syndrome cohort advocates a precision medicine approach to disease management. Kidney Int 91:937-947. https://doi.org/10.1016/j.kint.2016.10.013

2. Ibarra A, Hetzer MW (2015) Nuclear pore proteins and the control of genome functions. Genes Dev 29:337-349. https://doi.org/10.1101/gad.256495.114

3. Gee HY, Sadowski CE, Aggarwal PK, Porath JD, Yakulov TA, Schueler M, Lovric S, Ashraf S, Braun DA, Halbritter J, Fang H, Airik R, Vega-Warner V, Cho KJ, Chan TA, Morris LG, ffrench-Constant C, Allen N, McNeill H, Buscher R, Kyrieleis H, Wallot M, Gaspert A, Kistler T, Milford DV, Saleem MA, Keng WT, Alexander SI, Valentini RP, Licht C, Teh JC, Bogdanovic R, Koziell A, Bierzynska A, Soliman NA, Otto EA, Lifton RP, Holzman LB, Sibinga NE, Walz G, Tufro A, Hildebrandt F (2016) FAT1 mutations cause a glomerulotubular nephropathy. Nat Commun 7:10822. https://doi.org/10.1038/ncomms10822

4. Prasad R, Hadjidemetriou I, Maharaj A, Meimaridou E, Buonocore F, Saleem M, Hurcombe J, Bierzynska A, Barbagelata E, Bergada I, Cassinelli H, Das U, Krone R, Hacihamdioglu B, Sari E, Yesilkaya E, Storr HL, Clemente M, Fernandez-Cancio M, Camats N, Ram N, Achermann JC, Van Veldhoven PP, Guasti L, Braslavsky D, Guran T, Metherell LA (2017) Sphingosine-1-phosphate lyase mutations cause primary adrenal insufficiency and steroid-resistant nephrotic syndrome. J Clin Invest 127:942-953. https://doi.org/10.1172/JCI90171

5. Ibarra A, Benner C, Tyagi S, Cool J, Hetzer MW (2016) Nucleoporin-mediated regulation of cell identity genes. Genes Dev 30:2253-2258. https://doi.org/10.1101/gad.287417.116

6. Grandi P, Dang T, Pane N, Shevchenko A, Mann M, Forbes D, Hurt E (1997) Nup93, a vertebrate homologue of yeast Nic96p, forms a complex with a novel 205-kDa protein and is required for correct nuclear pore assembly. Mol Biol Cell 8:2017-2038. https://doi.org/10.1091/mbc.8.10.2017

7. Braun DA, Lovric S, Schapiro D, Schneider R, Marquez J, Asif M, Hussain MS, Daga A, Widmeier E, Rao J, Ashraf S, Tan W, Lusk CP, Kolb A, Jobst-Schwan T, Schmidt JM, Hoogstraten CA, Eddy K, Kitzler TM, Shril S, Moawia A, Schrage K, Khayyat AIA, Lawson JA, Gee HY, Warejko JK, Hermle T, Majmundar AJ, Hugo H, Budde B, Motameny S, Altmuller J, Noegel AA, Fathy HM, Gale DP, Waseem SS, Khan A, Kerecuk L, Hashmi S, Mohebbi N, Ettenger R, Serdaroglu E, Alhasan KA, Hashem M, Goncalves S, Ariceta G, Ubetagoyena M, Antonin W, Baig SM, Alkuraya FS, Shen Q, Xu H, Antignac C, Lifton RP, Mane S, Nurnberg P, Khokha MK, Hildebrandt F (2018) Mutations in multiple components of the nuclear pore complex cause nephrotic syndrome. J Clin Invest 128:4313-4328. https://doi.org/10.1172/JCI98688

8. Bierzynska A, Soderquest K, Dean P, Colby E, Rollason R, Jones C, Inward CD, McCarthy HJ, Simpson MA, Lord GM, Williams M, Welsh GI, Koziell AB, Saleem MA, NephroS, Syndrome UKsoN (2017) MAGI2 Mutations Cause Congenital Nephrotic Syndrome. J Am Soc Nephrol 28:1614-1621. https://doi.org/10.1681/ASN.2016040387

9. Zhang X, Chen S, Yoo S, Chakrabarti S, Zhang T, Ke T, Oberti C, Yong SL, Fang F, Li L, de la Fuente R, Wang L, Chen Q, Wang QK (2008) Mutation in nuclear pore component NUP155 leads to atrial fibrillation and early sudden cardiac death. Cell 135:1017-1027. https://doi.org/10.1016/j.cell.2008.10.022

10. Dorval G, Kuzmuk V, Gribouval O, Welsh GI, Bierzynska A, Schmitt A, Miserey-Lenkei S, Koziell A, Haq S, Benmerah A, Mollet G, Boyer O, Saleem MA, Antignac C (2019) TBC1D8B Loss-of-Function Mutations Lead to X-Linked Nephrotic Syndrome via Defective Trafficking Pathways. Am J Hum Genet 104:348-355. https://doi.org/10.1016/j.ajhg.2018.12.016

11. Hashimoto T, Harita Y, Takizawa K, Urae S, Ishizuka K, Miura K, Horita S, Ogino D, Tamiya G, Ishida H, Mitsui T, Hayasaka K, Hattori M (2019) In Vivo Expression of NUP93 and Its Alteration by NUP93 Mutations Causing Focal Segmental Glomerulosclerosis. Kidney Int Rep 4:1312-1322. https://doi.org/10.1016/j.ekir.2019.05.1157

12. Braun DA, Sadowski CE, Kohl S, Lovric S, Astrinidis SA, Pabst WL, Gee HY, Ashraf S, Lawson JA, Shril S, Airik M, Tan W, Schapiro D, Rao J, Choi WI, Hermle T, Kemper MJ, Pohl M, Ozaltin F, Konrad M, Bogdanovic R, Buscher R, Helmchen U, Serdaroglu E, Lifton RP, Antonin W, Hildebrandt F (2016) Mutations in nuclear pore genes NUP93, NUP205 and XPO5 cause steroid-resistant nephrotic syndrome. Nat Genet 48:457-465. https://doi.org/10.1038/ng.3512

13. Galy V, Mattaj IW, Askjaer P (2003) Caenorhabditis elegans nucleoporins Nup93 and Nup205 determine the limit of nuclear pore complex size exclusion in vivo. Mol Biol Cell 14:5104-5115. https://doi.org/10.1091/mbc.e03-04-0237

14. Weavers H, Prieto-Sanchez S, Grawe F, Garcia-Lopez A, Artero R, Wilsch-Brauninger M, Ruiz-Gomez M, Skaer H, Denholm B (2009) The insect nephrocyte is a podocyte-like cell with a filtration slit diaphragm. Nature 457:322-326. https://doi.org/10.1038/nature07526

15. Zhuang S, Shao H, Guo F, Trimble R, Pearce E, Abmayr SM (2009) Sns and Kirre, the Drosophila orthologs of Nephrin and Neph1, direct adhesion, fusion and formation of a slit diaphragm-like structure in insect nephrocytes. Development 136:2335-2344. https://doi.org/10.1242/dev.031609

16. Seelow D, Schuelke M, Hildebrandt F, Nurnberg P (2009) HomozygosityMapper--an interactive approach to homozygosity mapping. Nucleic Acids Res 37 (Web Server issue):W593-W599. https://doi.org/10.1093/nar/gkp369

17. Rodrigues CH, Pires DE, Ascher DB (2018) DynaMut: predicting the impact of mutations on protein conformation, flexibility and stability. Nucleic Acids Res 46:W350-W355. https://doi.org/10.1093/nar/gky300

18. Jumper J, Evans R, Pritzel A, Green T, Figurnov M, Ronneberger O, Tunyasuvunakool K, Bates R, Zidek A, Potapenko A, Bridgland A, Meyer C, Kohl SAA, Ballard AJ, Cowie A, Romera-Paredes B, Nikolov S, Jain R, Adler J, Back T, Petersen S, Reiman D, Clancy E, Zielinski M, Steinegger M, Pacholska M, Berghammer T, Bodenstein S, Silver D, Vinyals O, Senior AW, Kavukcuoglu K, Kohli P, Hassabis D (2021) Highly accurate protein structure prediction with AlphaFold. Nature 596:583-589. https://doi.org/10.1038/s41586-021-03819-2

19. McLaren W, Gil L, Hunt SE, Riat HS, Ritchie GR, Thormann A, Flicek P, Cunningham F (2016) The Ensembl Variant Effect Predictor. Genome Biol 17:122. https://doi.org/10.1186/s13059-016-0974-4

20. Frazer J, Notin P, Dias M, Gomez A, Min JK, Brock K, Gal Y, Marks DS (2021) Disease variant prediction with deep generative models of evolutionary data. Nature 599 :91-95. https://doi.org/10.1038/s41586-021-04043-8
