# Supplementary material for: Exploring the relevance of NUP93 variants in steroid-resistant nephrotic syndrome using next generation sequencing and a fly kidney model
Source: Pediatr Nephrol. 2022 Feb 24;37(11):2643–56. doi: 10.1007/s00467-022-05440-5 (PMC9489583; doi:10.1007/s00467-022-05440-5)
Supplement: Supplementary file 2 — Supplementary file2 (PPTX 1.14 MB) [file 467_2022_5440_MOESM2_ESM.pptx]

## Slide 1
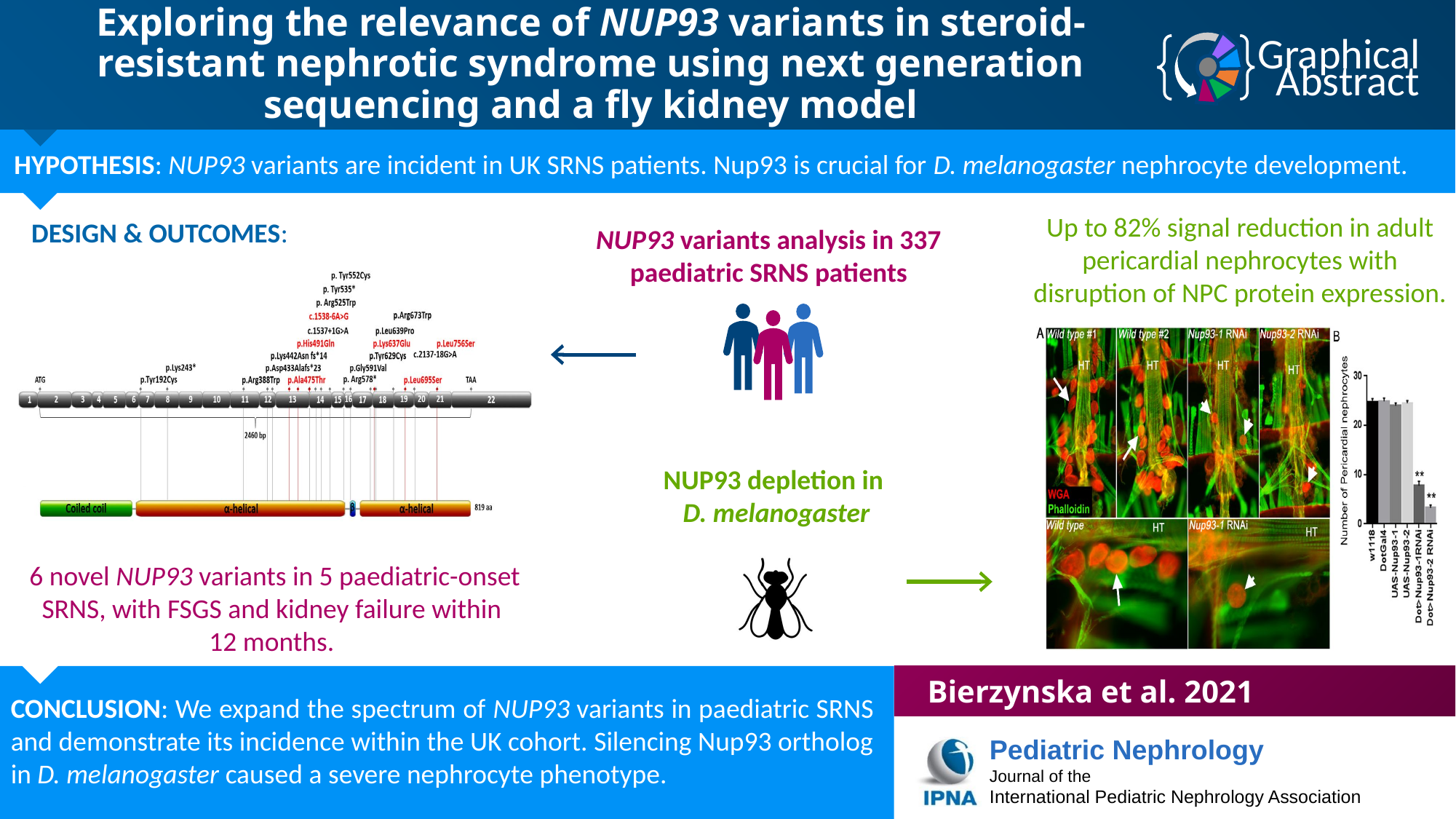

Exploring the relevance of NUP93 variants in steroid-resistant nephrotic syndrome using next generation sequencing and a fly kidney model
HYPOTHESIS: NUP93 variants are incident in UK SRNS patients. Nup93 is crucial for D. melanogaster nephrocyte development.
Up to 82% signal reduction in adult pericardial nephrocytes with disruption of NPC protein expression.
DESIGN & OUTCOMES:
NUP93 variants analysis in 337 paediatric SRNS patients
NUP93 depletion in
D. melanogaster
6 novel NUP93 variants in 5 paediatric-onset SRNS, with FSGS and kidney failure within
12 months.
Bierzynska et al. 2021
CONCLUSION: We expand the spectrum of NUP93 variants in paediatric SRNS and demonstrate its incidence within the UK cohort. Silencing Nup93 ortholog in D. melanogaster caused a severe nephrocyte phenotype.
